# Magnesium Fertilization Affected Rice Yields in Magnesium Sufficient Soil in Heilongjiang Province, Northeast China

**DOI:** 10.3389/fpls.2021.645806

**Published:** 2021-05-11

**Authors:** Zhilei Liu, Qiuhong Huang, Xiaohui Liu, Pengfei Li, Muhammad Rehman Naseer, Yeqi Che, Yaning Dai, Xiangyu Luo, Donghui Liu, Lili Song, Baiwen Jiang, Xianlong Peng, Cailian Yu

**Affiliations:** ^1^College of Resources and Environment, Northeast Agricultural University, Harbin, China; ^2^Key Laboratory of Black Soil Utilization and Protection, Heilongjiang Province, Harbin, China; ^3^Key Laboratory of Germplasm Innovation, Physiology and Ecology of Grain Crop in Cold Region (Northeast Agricultural University), Ministry of Education, Harbin, China; ^4^Institute of Chemical and Environmental Engineering, Harbin University of Science and Technology, Harbin, China

**Keywords:** soil exchangeable Mg, paddy soil, soil application, foliar application, *Oryza sativa*, yield

## Abstract

Magnesium (Mg) plays a crucial role in rice yield. Heilongjiang Province is the main rice-producing region of China, playing an important role in guaranteeing China's and the world's grain security. However, rarely Mg fertilization is applied in this province. Soil Mg status of main rice-producing areas in Heilongjiang Province was investigated and Mg fertilizer experiments were conducted aiming to provide fertilizer recommendation in this region. A total of 358 soil samples from the 0–20 cm and 20–40 cm soil layer from the main rice-producing areas of Heilongjiang Province were collected to analyze soil exchangeable Mg (ex-Mg) and relative chemical properties. Meanwhile, field experiments of soil and foliar Mg application were performed in 2017–2019 to identify the effect of this nutrient on rice yield. The results showed that the ex-Mg concentration in the 0–20 cm and 20–40 cm soil layer was 282 mg kg^−1^ and 243 mg kg^−1^, respectively. Moreover, ex-Mg ranged on the abundant and exceptionally abundant level accounted for 75% in 0–20 cm and 55.3% in 20–40 cm. The ex-Mg concentration in the upper soil layer was higher than in the lower soil layer and varied depending on regions, which the west part of Heilongjiang Province showed the highest concentration in both soil layers. Correlation analysis showed that there had a significant (*P* < 0.05) linear relationship between ex-Mg and pH, CEC, ex-K, Ca, K/Mg, and Ca/Mg. Meanwhile, the results of path coefficients demonstrated that pH, CEC, and Ca/Mg had the most direct effect on ex-Mg concentration among these above factors. Soil Mg application had little effect on rice yield, which might be related to the soil Mg concentration and availability, and root uptake activity. Foliar Mg application increased rice yield by 8.45% (*P* < 0.05) compared to without Mg treatment, increased 1,000-grain weight by 2.62% (*P* < 0.05), and spikelet number per panicle by 4.19% (*P* < 0.05). In general, the paddy soil ex-Mg concentration in Heilongjiang Province was abundant. Soil-applied Mg played little role in rice yield in ex-Mg abundant regions, while foliar application increased rice yields significantly via increasing 1,000-grain weight and spikelet number per panicle.

## Introduction

Magnesium (Mg) is the fourth most abundant nutrient element after nitrogen (N), phosphorus (P), and potassium (K), has irreplaceable effects on the crop physiology and plays key roles in plant defense mechanisms to abiotic stress (Cakmak and Kirkby, 2008; Cakmak and Yazici, 2010; Cakmak, 2013; Mengutay et al., 2013; Senbayram et al., 2015). Mg is the main component of chlorophyll and has a high impact on photosynthesis, enzyme activation, and the formation and utilization of ATP. Furthermore, Mg plays an essential role in phloem loading and transport of photoassimilates into sink organs, mainly at the grain filling stage, which is crucial for grain yields (Cakmak et al., 1994; Hermans et al., 2005; Ruan et al., 2012). Consequently, maintaining sufficient Mg availability is important for plant growth.

Mg is mainly obtained from the soil through the root system. Therefore, only sufficient soil Mg can ensure the supply of this nutrient for plant growth. There are four different Mg fractions (water-soluble, exchangeable, fixed, and organic) in soils, while for typical agronomic soils, an extensive amount of total soil Mg is bound in an exchangeable form (Gransee and FüHrs, 2013). However, the amounts of exchangeable Mg (ex-Mg) that are released from soils are generally small in comparison with the amounts needed to sustain high crop yields and quality (Cakmak and White, 2020). In addition to an absolute lack of Mg in soil, rainfall, soil acidification, high soil CEC or calcium ion (Ca^2+^) and other factors cause Mg leaching loss (Gransee and FüHrs, 2013). Moreover, due to continuous high crop yields, increasingly more Mg is taken away from the soil by the harvest of grains and straws. Unreasonable fertilization, especially the excessive application of N and K fertilizers, also inhibits the absorption of Mg by roots due to competition between ions (Senbayram et al., 2015; Xie et al., 2020). Consequently, Mg deficiency or potential deficiency is becoming increasingly common worldwide. According to reports, 63.6% of the arable soil in China is severely deficient or deficient in Mg (Wang, 2020).

Mg deficiency in rice (*Oryza sativa* L.) is characterized by the occurrence of chlorosis between the ribs of older leaves, which spreads and can develop into necrosis. Plant growth is impaired, and mature leaf fall causes a drop in production (Dechen et al., 2015). Mg fertilization applications are critically essential for crops with high Mg concentrations, yields, and production quality (Senbayram et al., 2015; Zhang et al., 2020). A meta-analysis of 570 paired observations retrieved from different crop systems was performed to analyze the effect of Mg fertilization on crop production, and the effect of Mg fertilization on yield was 9.4% under conditions of severe Mg deficiency (ex-Mg < 60 mg kg^−1^) (Wang et al., 2020). Mg deficiency may occur even if sufficient or higher environmental Mg conditions exist, which is related to Mg availability (Gransee and FüHrs, 2013; Grzebisz, 2013). Studies have shown that Mg applications can increase crop yields in Mg-sufficient soils to some extent as well (Wang et al., 2020). Consequently, how to fertilize reasonably to improve Mg availability is a problem worthy of study in soil Mg-rich regions.

Soil application is an ancient and normal fertilization practice, which is the main approach for Mg fertilizer supplementation (BratašEvec et al., 2013; Ceylan et al., 2016). However, Mg deficiency destroys crop phloem transport tissue, promotes lignification, and constantly wakes the ability of the root system to absorb nutrients (Cakmak, 2013; Guo et al., 2016). Therefore, soil fertilization cannot replenish Mg in time in this situation. Nutrient application by foliar methods has a direct effect on the mineral concentrations of the elements used and has a more direct effect on growth parameters and crop response to fertilizer application when compared with soil application (Cakmak and Kirkby, 2008; Rehman et al., 2018). Foliar application of Mg can promote photosynthesis, prevent root pre-mature senility, increase tissue Mg concentrations and improve crop yields and quality (Ceylan et al., 2016; Rehman et al., 2018). However, some studies have demonstrated that due to the high concentrations of K and Ca in soil, nutrient imbalances induce Mg deficiency in citrus [*Poncirus trifoliata* (L.) Raf.] and grapes (*Vitis vinifera* L.), so it was difficult to increase leaves Mg content by soil or foliar application of Mg fertilizer (Morton et al., 2008; Trolove et al., 2008). Thus, it can be seen that applying Mg fertilizers scientifically and rationally is essential for high crop yields and quality under different situations, such as Mg-rich soil, low soil pH, or high soil CEC.

Heilongjiang, locating in northeast China, is the largest single rice-producing region of China and plays important role in guaranteeing the grain security of China. However, there are few Mg fertilizer applications in Heilongjiang according to an investigation of rice fertilizer application practices, as was revealed in our previous investigation (data are not shown). To identify the contribution of Mg to rice yield, soil ex-Mg concentrations in the main paddy rice-producing regions of Heilongjiang Province were investigated, and field experiments of Mg fertilizer were conducted. This research mainly identified paddy soil ex-Mg concentrations in Heilongjiang and the effect of Mg fertilizer application on rice yield. The results of this study could provide guidance for Mg fertilization in Mg-rich paddy soil.

## Materials and Methods

### Study Area

Heilongjiang Province is located in northeast China between 43°26′-53°33′N and 121°11′-135°05′E where the Songhua River, Wusuli River, Suifen River, and Heilong River flow through, covering an area of 473,000 km^2^. It has a humid and semi-humid monsoon climate of the cold temperate zones, which belongs to the Dwa, Dwb, and Dwc climate region according to Koppen–Geiger climate classification system (Peel et al., 2007; Li et al., 2018). The average annual temperature is ~0.13–6.01°C, the average annual precipitation is 380–600 mm, and the total sunshine amount is 2,590 h during the rice-growing season. The rice planting area in Heilongjiang was ~3.78 million hectares in 2018, which was 12.8% of total China. The main rice-producing areas of Heilongjiang Province are divided into three regions according to their geographical location: the Songnen Plain (western part), south-central part, and Sanjiang Plain (eastern part). Among them, the Sanjiang Plain is the most extensive rice area and is also one of the major rice-producing regions of China. The soil types are black soil (Luvic Phaeozem, FAO); chernozem (Haplic Chernozem, FAO); and Baijang soil (Albic Luvisols, FAO).

### Soil and Plant Sampling and Determination

**Figure 1:**
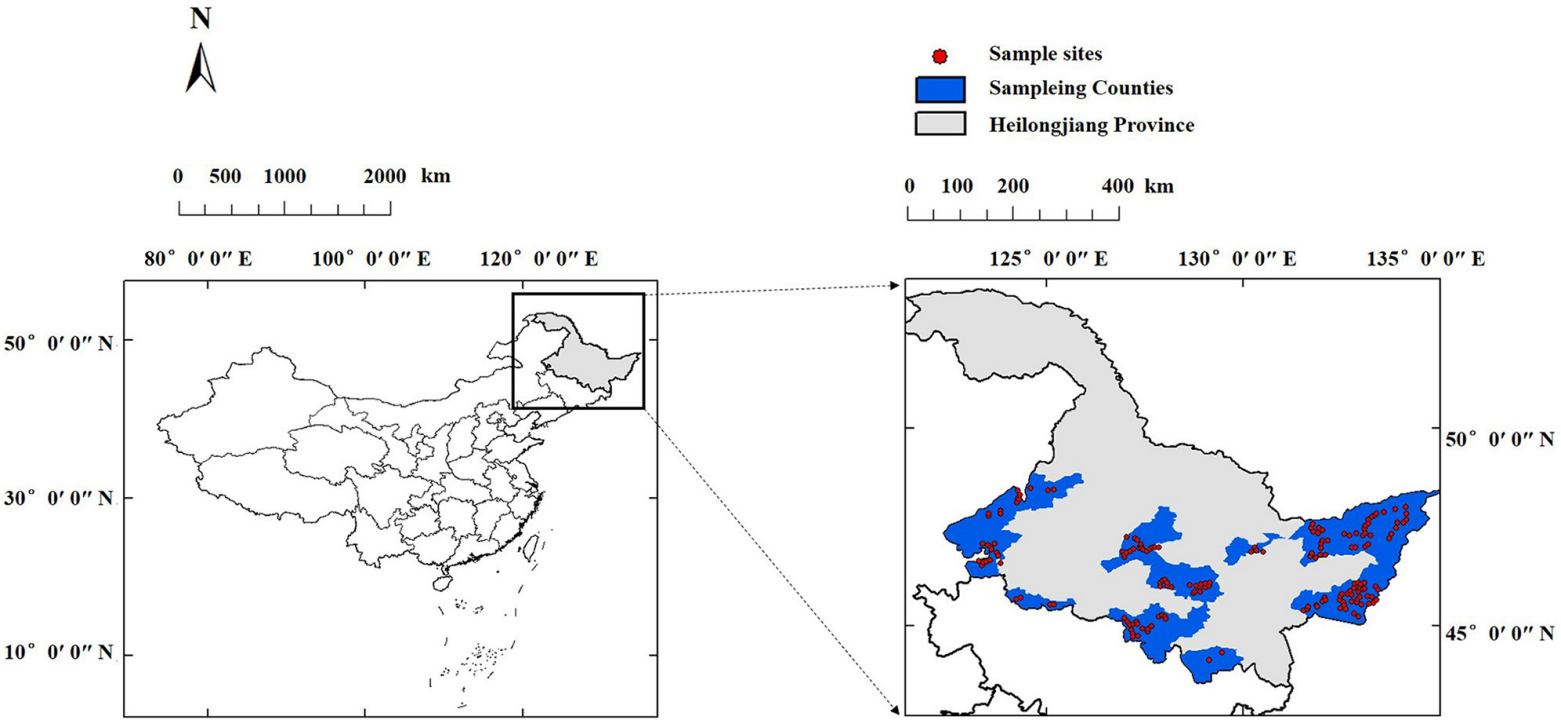
Soil sampling locations in Heilongjiang Province, Northeast China. *n* = 179.

To investigate the distribution of soil ex-Mg in Heilongjiang Province, soil samples were collected in 2016. The sampling sites of this study were distributed in the main rice-producing areas of the Songnen Plain, south-central area, and Sanjiang Plain in Heilongjiang Province and included 23 counties. GPS was used to determine the longitude and latitude of the sampling plots, and a soil sample was collected every 100 km^2^. The sampling depths were 0–20 cm and 20–40 cm, and a total of 358 representative soils was collected. Soil sampling plots were randomly chosen in each typical zone with five replicates and were homogenized by hand mixing with a mixed soil sample of ~1 kg. The collected soil samples were brought back to the laboratory and placed in a dark, ventilated environment until the soil samples dried. The soil samples were ground, sieved to an 80-mesh size, mixed well, and then sealed for later analysis. The locations of the sampling sites are shown in Figure 1.

A glass electrode was used to calculate soil pH levels by using a ratio of 1:2.5 soil/water suspensions. 1 M NH_4_Cl–NH_4_OAc was used to extract exchangeable K (Knudsen et al., 1982). CEC was derived by substitution of exchangeable cations with 1 M NH_4_Cl-NH_4_OAc (Thomas, 1982). The ex-Ca and ex-Mg in soil were extracted using 1 M NH_4_Cl-NH_4_OAc (Chapman, 1965).

### Field Experiments

To identify the effects of Mg on rice yields in Heilongjiang Province, field experiments were conducted at two sites during 2017–2019. The two sites, Daxing (DX) and Wuchang (WC) are located in the Sanjiang Plain and south-central areas, respectively. Prior to experimentation, soil samples were extracted from the upper 20 cm layer for chemical analysis, and the results are shown in Table 1.

**Table 1:** Soil chemical fertility properties.

**Site**	**O. M. (g kg**^**−1**^**)**	**Total N (g kg**^**−1**^**)**	**Olsen P (mg kg**^**−1**^**)**	**Available K (mg kg**^**−1**^**)**	**Available Mg (mg kg**^**−1**^**)**	**pH**
WC	43.5	2.16	15.4	138	255	5.70
DX1	33.6	1.65	21.6	134	265	6.11
DX2	37.7	1.90	28.0	208	251	6.02

In 2017, two nutrient management treatments were set in WC: (1) Optimized NPK fertilizer (NPK) and (2) soil Mg application based on NPK (NPK+SMg). In 2018, three treatments, namely, NPK, NPK+SMg, and foliar Mg application based on NPK (NPK+FMg), were used in WC. Two treatments, namely, NPK and NPK+FMg, were used at two sites (e.g., DX1 and DX2) in DX in 2018 and at one site in 2019. Detailed information for the fertilizer application rates and timing of each treatment is shown in Table 2. Soil applications of Mg fertilized with magnesium oxide (MgO) 18 kg hm^−2^ as the basal dose, and the fertilizer used magnesium sulfate monohydrate (MgSO_4_·H_2_O). Foliar Mg was applied at a MgO concentration of 0.34 kg hm^−2^ at the jointing stage, and the fertilizer was magnesium sulfate heptahydrate (MgSO_4_·7H_2_O). The N, P, and K fertilizers were urea, calcium superphosphate, and potassium chloride, respectively. Basal fertilizer was sprinkled by hand 10 d before transplanting, and the top-dressing fertilizer was also sprinkled into the water by hand.

**Table 2:** Timing and amounts of applied fertilizer (kg hm^−2^).

**Year**	**Site**	**Treatment**	**Total fertilizer rate (N-P_**2**_O_**5**_-K_**2**_O)**	**Basal fertilizer (N-P_**2**_O_**5**_-K_**2**_O)**	**Tillering fertilizer (N-P_**2**_O_**5**_-K_**2**_O)**	**Panicle fertilizer (N-P_**2**_O_**5**_-K_**2**_O-MgO)**
2017/2018	WC	NPK	100-40-80	40-40-55	30-0-0	30-0-25-0
		NPK+SMg	100-40-80	40-40-55	30-0-0	30-0-25-18
		NPK+FMg	100-40-80	40-40-55	30-0-0	30-0-25-0.34
2018	DX	NPK	95-46-60	45-46-30	30-0-0	20-0-30-0
		NPK+FMg	95-46-60	45-46-30	30-0-0	20-0-30-0.34
2019	DX	NPK	105-50-60	55-50-30	30-0-0	20-0-30-0
		NPK+FMg	105-50-60	55-50-30	30-0-0	20-0-30-0.34

*NPK, Optimized N, P, K fertilizer treatment; SMg, soil Mg application; FMg, foliar Mg application*.

The plots were ~400 m^2^ (20 × 20 m) and were arranged in a randomized complete block design with three replicates per treatment. The rice varieties, “Wouyoudao-4” and “Sanjiang-6,” which are widely grown in Heilongjiang Province, were grown in WC, DX, respectively. In 2017–2019, pregerminated seeds were sown in a nursery bed on 10–13 April, and the 3.5-leaf-stage seedlings were transplanted on 10–16 May with a hill spacing of 36 × 22 cm in WC and 30 × 13 cm in DX with 3–5 seedlings per hill. At maturity, grain yields were determined from a 2 m^2^ area and were adjusted to a moisture content of 0.14 kg H_2_O kg^−1^ fresh weight.

### Statistical and Geostatistical Methods

Statistical analyses were performed using SPSS 20.0. Correlation and path analysis was used to assess the relationship between ex-Mg concentrations and soil properties, and significance levels of *P* < 0.05 and *P* < 0.01 were used.

One-way ANOVA was used for the field experiment. Significant differences between individual mean values were determined using Duncan's test at the 5% confidence level and were based on three biological replicates for each treatment. The values in the table represent the means ± standard errors.

The kriging interpolation technique was applied to calculate the ex-Mg concentrations of the main rice-producing areas in Heilongjiang Province. Prior to the interpolation, a geostatistical method tool in the ArcGIS 10.0 software was used to conduct data analyses, including data distribution, outlier identification, and trend analyses.

The semivariogram is half of the expected squared difference between paired data values *z*(*x*) and *z*(*x*+*h*) to the lag distance *h*, by which locations are separated (Webster and Oliver, 2001). For discrete sampling sites, such as those in this study, the function is usually written in the form:

$$r\left(h\right)=\frac{1}{2N\left(h\right)}\sum_{i=1}^{N\left(h\right)}\left[z(x_{i}-z(x_{i}+h\right){]}^{2}$$

where *z*(*x*_*i*_) is the value of the variable *z* at location *x*_*i*_ and *N*(*h*) is the number of pairs of sample points separated by *h*.

The experimental variogram is calculated for several lag distances. Kriging is considered to be an ideal technique for spatial forecasting. It is a theoretical weighted moving average:

$$\begin{array}{l} \hat{z}\left(x_{0}\right)=\overset{n}{\underset{i=1}{\sum }}\lambda_{i}z\left(x_{i}\right) \end{array}$$

where $\hat{z}(x_{0})$ is the value to be estimated at location *x*_0_, *z*(*x*_*i*_) is the known value at sampling site *x*_*i*_ and *n* is the number of sites within the search neighborhood used for the estimation.

## Results

### Ex-Mg Concentration Frequency Distribution

**Figure 2:**
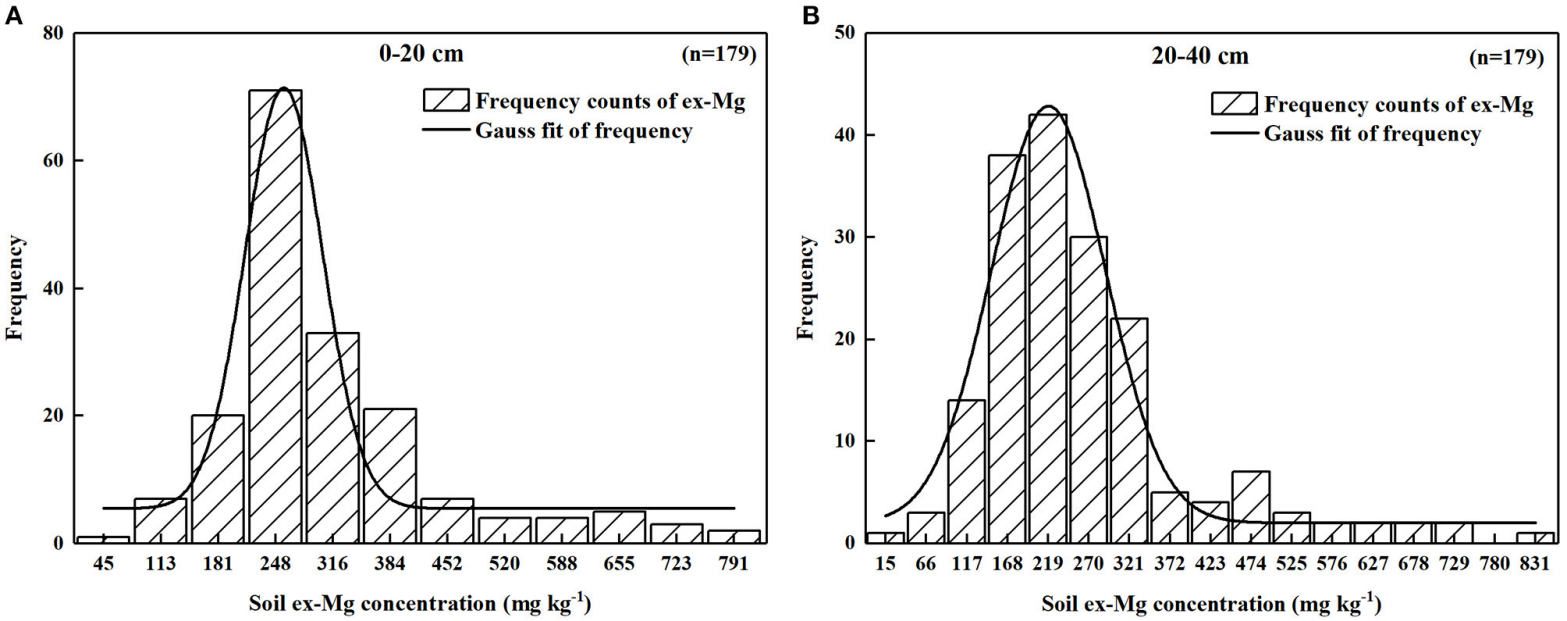
**(A,B)** Histograms of paddy soil ex-Mg concentrations of in Heilongjiang Province, Northeast China.

The histograms of paddy soil exchangeable Mg concentrations with the normal distribution curves in the 0–20 cm and 20–40 cm soil layers in Heilongjiang Province are shown in Figure 2. The soil exchangeable Mg concentrations were mainly concentrated on the left side of the average value, and the axis of symmetry also appeared to the left of the average value, which indicated that there was a specific variability in Mg concentrations both in the 0–20 cm and 20–40 cm soil layers in Heilongjiang paddy soils. However, the degree of the exchangeable Mg variations in the 20–40 cm soil layer was slightly less than those in the 0–20 cm soil layer, and there was a distribution characteristic of shifting to the left.

### Ex-Mg Concentration Vertical and Spatial Distribution

**Figure 3:**
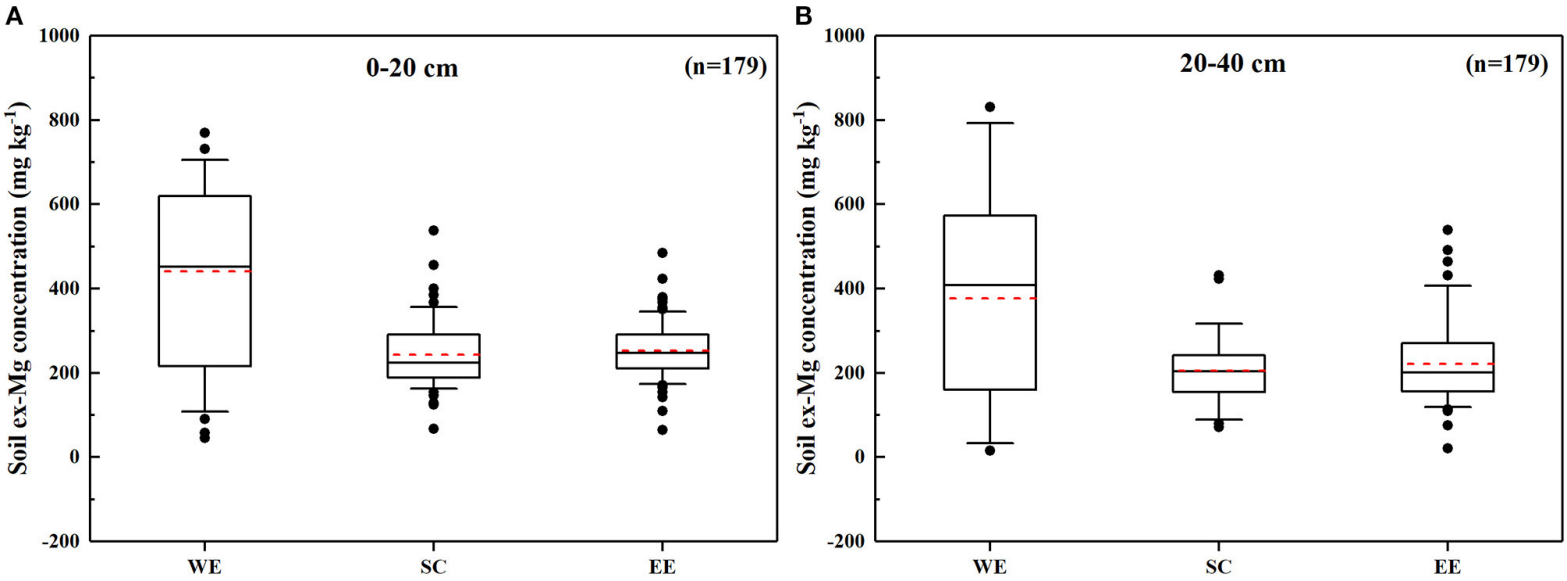
Paddy soil ex-Mg concentrations of the **(A)** 0–20 cm and **(B)** 20–40 cm soil layers in different regions of Heilongjiang Province. Solid black and dashed red lines indicate the median and mean, respectively. The box boundaries indicate the 75% and 25% quartiles; error bars indicate the 90th and 10th percentiles; the black dots indicate extreme values. WE, western part; SC, south-central part; EE, eastern part.

The vertical distribution of ex-Mg in the main rice-producing areas is shown in Figure 3. The ex-Mg concentration of paddy fields for the whole province was 15–1,130 mg kg^−1^, and the Mg concentrations were different between the 0–20 cm and 20–40 cm soil layers, for which the upper layer soil Mg concentration was higher than that of the lower layer. The average ex-Mg concentrations in the 0–20 cm and 20–40 cm soil layers were 282 and 243 mg kg^−1^, respectively.

**Figure 4:**
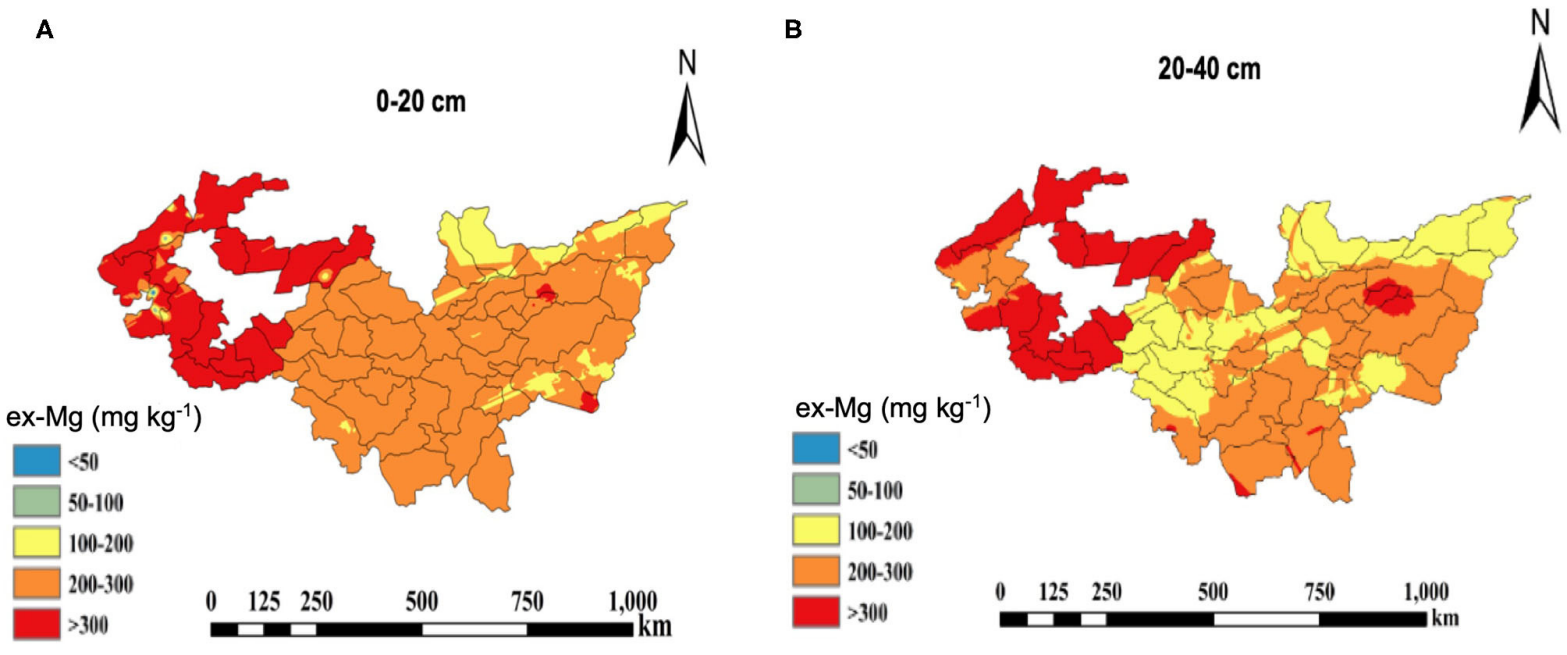
Spatial distribution map of ex-Mg in the 0–20 cm **(A)** and 20–40 cm **(B)** paddy soil layers in Heilongjiang Province.

The spatial distribution map of ex-Mg in Heilongjiang paddy soils is shown in Figure 4. A clear variation pattern of ex-Mg concentrations can be observed more intuitively in this figure. Within the 0–20 cm soil layer, the ex-Mg contents in the Songnen Plain were the highest at 441 mg kg^−1^ on average and were followed by the Sanjiang Plain and south-central region at 252 and 243 mg kg^−1^, respectively (Figures 3A, 4A). The areas with high ex-Mg concentrations were mainly concentrated in Daqing City, Qiqihar City, and Youyi County of Shuangyashan City. There was a small area with ex-Mg concentrations below 100 mg kg^−1^. However, areas with ex-Mg concentrations >200 mg kg^−1^ accounted for more than 95% of the whole area. In the 20–40 cm soil layer, the ex-Mg concentrations in the paddy soils of Heilongjiang Province showed a trend that was similar to that in 0–20 cm layer, in which the highest concentrations were present in the western part, and the lowest concentrations were present in the central part (Figures 3B, 4B). Similar to the 0–20 cm soil layer, there was a small region with ex-Mg concentrations below 100 mg kg^−1^, and regions with ex-Mg concentrations >200 mg kg^−1^ occupied more than 93% of the entire area. The areas with high ex-Mg concentrations were mainly concentrated in Daqing City, Qiqihar City, and Youyi County of Shuangyashan City as well.

According to the ex-Mg nutrient status, soil ex-Mg is divided into five grades: the exceptionally lacking Mg grade has <50 mg kg^−1^, the lacking grade has 50–100 mg kg^−1^, 100–200 mg kg^−1^ is medium grade, 200–300 mg kg^−1^ is abundant grade, and more than 300 mg kg^−1^ is the exceptionally abundant grade. Among all soil samples taken, soils at the abundant and exceptionally abundant levels accounted for 47.5% and 28.5% in the 0–20 cm soil layer, respectively; the medium level accounted for 21.2%, and soils at the lacking and exceptionally lacking levels accounted for only 2.8%. In the 20–40 cm soil layer, soils at the abundant and exceptionally abundant levels accounted for 34.1 and 21.2%, respectively; the medium and low soil levels accounted for 38 and 6.7%, respectively. Hence, soils in the paddy fields of Heilongjiang are rich in Mg.

### Correlation Analysis Between Soil Ex-Mg and Chemical Properties

**Figure 5:**
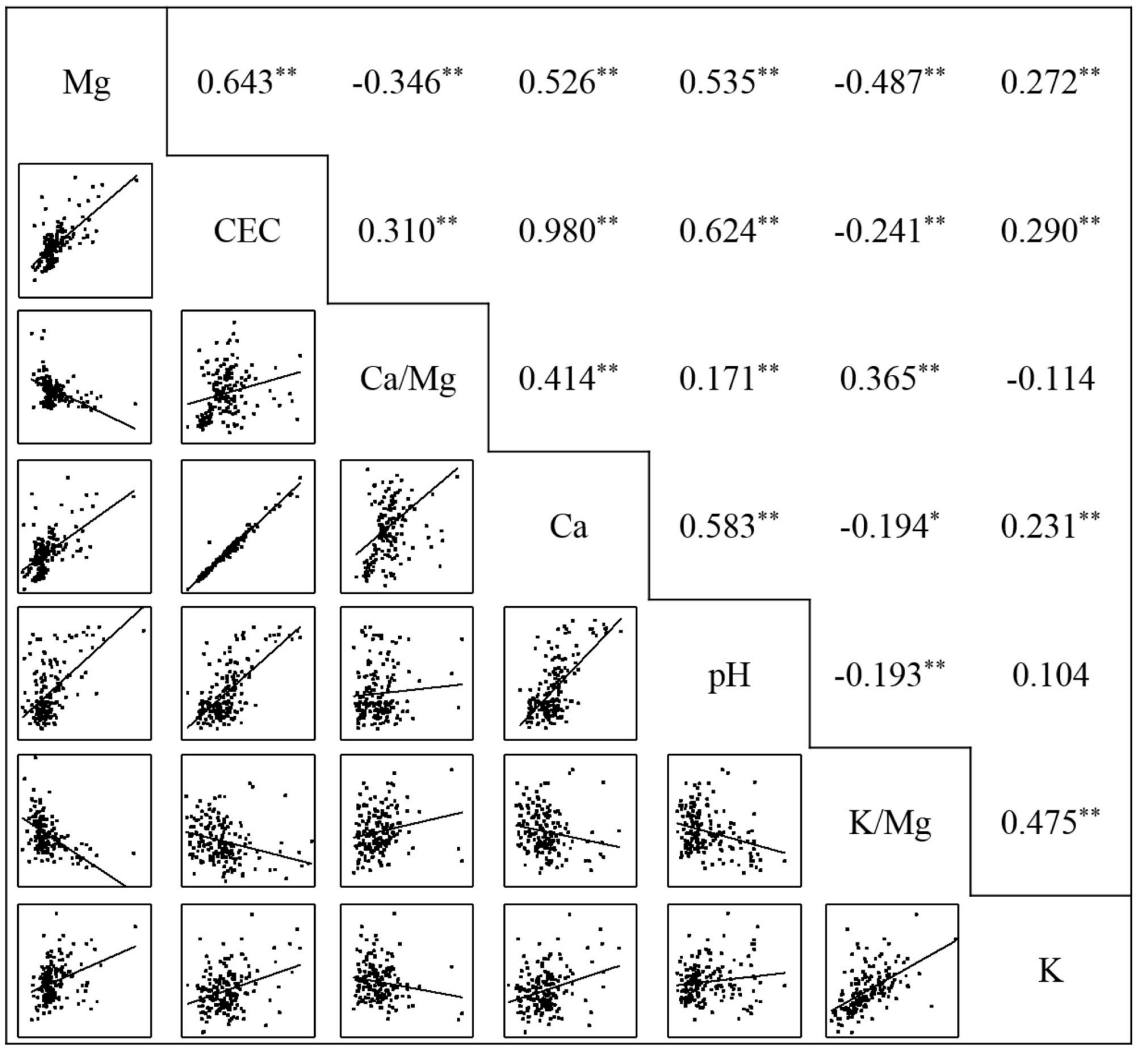
Correlation matrix among soil ex-Mg, pH, and soil properties. The values of the 0–20 cm soil layer were used (*n* = 179). ^*^ and ^**^ indicate that the corresponding values are significant at *P* < 0.05 and *P* < 0.01, respectively.

To clarify the relationship between soil ex-Mg and the main soil chemical properties, the correlation between ex-Mg concentration and chemical fertility indexes of the paddy soils (0–20 cm) in Heilongjiang was analyzed in this research (Figure 5). The results showed that there was a very significant positive correlation between ex-Mg concentration and soil pH, CEC, ex-K, and Ca, suggesting that soil pH, CEC, ex-K, and Ca were important factors for soil Mg concentrations, in which soil Mg concentration increased as these other factors increase. Furthermore, the results also showed that the Ca/Mg and K/Mg ratios were both negatively correlated with soil ex-Mg (*P* < 0.01), indicating that larger Ca/Mg or K/Mg ratios could reduce the availability of soil ex-Mg.

Path analysis was used to analyze the effects of soil ex-Ca, K, pH, CEC, Ca/Mg, and K/Mg on ex-Mg concentrations (Table 3). The results showed that the direct path coefficient of CEC was 2.007, while its indirect path coefficients via Ca/Mg and Ca were −0.118 and −1.355, respectively. The direct path coefficient of pH was 0.134, but its indirect path coefficient via CEC was 9.34 times its direct, and its indirect path coefficient via Ca was 0.806. The direct path coefficients of Ca/Mg and Ca were −0.380 and −1.383, respectively, exhibiting a negative effect on ex-Mg. However, the indirect path coefficients of Ca/Mg and Ca via CEC were 0.622 and 1.967, respectively, indicating that CEC not only affected ex-Mg directly but also indirectly affected ex-Mg.

**Table 3:** Path coefficients between soil properties and soil ex-Mg.

	**CEC**	**Ca/Mg**	**Ca**	**pH**	**K/Mg**	**Sum**
CEC	*2*.007	−0.118	−1.355	0.084	0.026	0.643
Ca/Mg	0.622	−0.380	−0.573	0.023	−0.039	−0.347
Ca	1.967	−0.157	−1.383	0.078	0.021	0.525
pH	1.252	−0.065	−0.806	*0*.134	0.021	0.536
K/Mg	−0.484	−0.139	0.268	−0.026	−0.107	−0.487

*The underlined data are direct path coefficients and the rest are indirect path coefficients, and the sum represents the sum of the direct and indirect path coefficients for each property*.

### Effect of Mg Fertilizer on Rice Yield

To identify the effects of Mg fertilizer on rice yields, three-year Mg fertilizer application experiments were conducted, and the results are given in Table 4. Soil Mg applications had little effect on rice yields in the 2-year experiments in comparison with NPK, while foliar Mg application increased rice yields by an average of 8.44% (*P* < 0.05) and 8.65% (*P* < 0.05) compared to the NPK treatments in WC and DX, respectively. In DX, Mg application via foliar spraying increased 1,000-grain weight by 2.62% (*P* < 0.05) on average compared to NPK in 2018 and 2019. In addition, foliar application of Mg increased spikelet number per panicle by 4.19% (*P* < 0.05) on average, whereas there was no significant difference in effective panicle number per unit and filling grain rate between the NPK and foliar Mg treatments.

**Table 4:** Rice yields and yield components for different nutrient management practices.

**Year**	**Site**	**Treatment**	**Spikelet number per panicle**	**Effective panicle number (m^**−2**^)**	**1,000-grain weight (g)**	**Grain-filling rate (%)**	**Yield (Mg hm^**−2**^)**
2017	WC	NPK	—	—	—	—	9.13 a
		NPK+SMg	—	—	—	—	9.12 a
2018	WC	NPK	70.87 b	445.90 a	30.67 a	69.44 a	6.93 ab
		NPK+SMg	72.92 b	405.60 b	30.69 a	71.36 a	6.75 b
		NPK+FMg	81.30 a	445.90 a	29.74 b	66.63 a	7.32 a
	DX1	NPK	83.24 a	615.00 a	25.33 b	79.40 a	10.27 b
		NPK+FMg	82.53 a	625.83 a	25.74 a	80.50 a	10.67 a
	DX2	NPK	66.85 b	661.21 a	23.53 b	89.90 a	10.03 b
		NPK+FMg	71.17 a	648.69 a	24.55 a	91.92 a	10.41 a
2019	DX2	NPK	76.71 b	522.57 a	26.33 b	68.61 a	6.51 b
		NPK+FMg	85.51 a	496.87 b	26.83 a	77.35 a	7.70 a

*NPK, Optimezed N, P, K fertilizer treatment; SMg, soil Mg application; FMg, foliar Mg application. Values are means ± standard errors of three biological replicates. Different letters following values within a column represent significant differences (P < 0.05) based on Duncan's test*.

## Discussion

The ex-Mg concentrations of Heilongjiang paddy fields in 0–20 cm and 20–40 cm soil layers were averaged 282 and 243 mg kg^−1^, respectively (Figure 3). Among all soil samples taken, soils at the abundant and exceptionally abundant levels accounted for 76% in the 0–20 cm soil layer and 55.3% in the 20–40 cm soil layer. Medium level accounted for 21.2% in the upper soil layer, which represents potentially Mg-deficient soil, and lower ex-Mg levels accounted for 2.8%. In general, Mg concentrations in the lower layer are usually higher than those in the upper layer because soil Mg easily leaches to the lower layer (Gransee and FüHrs, 2013). However, ex-Mg concentrations in the 0–20 cm soil layer were higher than those in the 20–40 cm layer in this research (Figure 3). This was mainly because the 0–20 cm layer of paddy soils in Heilongjiang Province is black soil or chernozem, which exhibits higher fertility. However, soils below 20 cm are mainly loess or sand due to the severe degradation of black soil in recent years and showed lower fertility (Liu et al., 2010). Moreover, the high Mg concentrations in the upper layer may also be related to the thickness of the plow pan of the paddy field. The paddy soil plow pan thickness in Heilongjiang is generally 13–18 cm (Pan et al., 2004), and Mg in the surface layer or upper layer may have difficulty leaching to the lower layer, which would result in Mg mainly accumulating in the 0–20 cm soil layer.

The ex-Mg concentrations in soils are greatly affected by pH values and CECs since ex-Mg is characterized by reversible binding of Mg to permanent and variable charges in soils, which are strongly dependent on soil pH (Gransee and FüHrs, 2013). Ex-Mg becomes non-exchangeable when the soil pH is lower than 6.0 and is subjected to leaching in acidic soils (Hailes et al., 1997; Wang et al., 2020). Mg is a relatively large molecule in comparison with the metal nutrient, such as copper (Cu) and zinc (Zn), which do not stick tightly to soil particles. Hence, even at high pH, Mg easily comes off of the soil particle and enters soil solution, thus have higher availability (Gransee and FüHrs, 2013; Mccauley et al., 2017). A similar result was obtained in this research that soil ex-Mg concentrations were highest when the soil pH was >7.5 (Supplementary Table 1). Meanwhile, the larger CEC, the stronger adsorption capacity of soil for exchangeable cations, which benefits ex-Mg accumulation in soil (Gransee and FüHrs, 2013). The path analysis also showed that CEC and pH had positive and highest direct effects on soil ex-Mg concentrations (Table 3), indicating that CEC and pH are the main factors that affected the Heilongjiang paddy soil Mg concentrations. The paddy fields in research regions are generally acidic, but the soil in the western part, such as the Songnen Plain, was mostly neutral or alkaline and had relatively higher CECs and pH. Consequently, the soil ex-Mg concentrations were higher in the Songnen Plain than in other regions (Figure 4).

The ratios of Ca, K, and Mg in the soil also affect the absorption of Mg by plants. For plants, an ideal soil has a Ca/Mg ratio of 6.5 and a K/Mg ratio of 0.5 (Kopittke and Menzies, 2007). In this research, the Ca/Mg and K/Mg ratios of the paddy soils in Heilongjiang were high, with mean values of 12.19 for Ca/Mg and 0.65 for K/Mg, and sites with Ca/Mg ratios >6.5 were counted for 85%, and a K/Mg ratio higher than 0.5 was observed in 71% (Supplementary Table 2). Among the three regions, although the average value of the K/Mg ratio was <5 only in the Songnen Plain, the Ca/Mg ratio in the Songnen Plain was the highest, with an average value of 15.22. The Ca/Mg and K/Mg ratios were negatively correlated with ex-Mg (Figure 5), and the direct path coefficient for Ca/Mg was −0.380 (Table 3), which demonstrated that the Ca/Mg ratio had a greater negative effect on soil Mg concentrations in Heilongjiang. Consequently, although the ex-Mg concentrations in the paddy fields of Heilongjiang are rich, the higher Ca/Mg and K/Mg ratios in some sites may inhibit the absorption of Mg by rice.

Excessive fertilization, especially ammonium fertilizer, is one of the main factors that cause soil Mg leaching and inhibit the absorption of Mg by crops (Gransee and FüHrs, 2013; Grzebisz, 2013). Our previous research found that although the amount of N fertilizer used in paddy fields in Heilongjiang was the lowest in China when compared with optimized crop management, the N fertilizer dosage used by farmers was still ~50% higher (Peng et al., 2015). Excessive N fertilizer not only causes the waste of N fertilizer but also increases antagonism between cations and inhibits the absorption of other ions, such as Mg^2+^ and K^+^ (Cakmak and Yazici, 2010; Boaretto et al., 2020). Consequently, balanced fertilization should increase the input of Mg fertilizer in production to prevent absolute Mg deficiency caused by long-term unbalanced crop fertilization practices that neglect soil Mg depletion through crop Mg removal (Van Der Pol and Traore, 1993; Wang et al., 2020). Mg fertilizer not only could mitigate the detrimental effects of abiotic stress on plants but improve crop yields for most production cases, particularly for Mg-deficient regions (Boaretto et al., 2020; Cakmak and White, 2020; Wang et al., 2020). Choudhury and Khanif (2001) estimated the rice yield response to Mg rates that rice yields were significantly positively correlated with Mg fertilizer application rates at both sites. The results of 3-year experiments showed that optimizing N fertilizer management could significantly increase rice yields in Mg-rich soil (Table 4). However, soil application of Mg fertilizer based on optimized N management had little effect on rice yields, which was contrary to what we expected. The possible reasons for these results are that Mg application to the soil causes a greater portion of the fertilizer to not reach the roots due to secondary soil interactions and leaching (Gransee and FüHrs, 2013; Chen et al., 2018). In addition, during the jointing stage of Mg application, although this is the period when rice requires the largest amounts of Mg, the decline of root activity in the later growing stage limits root Mg uptake even with excessive Mg in the soil (Mishra and Salokhe, 2011), which eventually leads to soil-applied Mg having a lesser effect.

Mg transport from soil to rice roots and tissues in the aboveground parts includes at least uptake, xylem loading and uploading processes, which is involved in lots of Mg transporters such as *OsMGT* and *OsMRS2* protein family (Chen et al., 2012; Chen and Ma, 2013; Saito et al., 2013). Plant stores Mg in great amounts within vacuoles, severing as a pool for maintaining Mg homeostasis in other cells during growth (Hauer-Jákli and Tränkner, 2019). Plants will transport Mg through the phloem from old tissues to young tissues when Mg is deficiency (Marschner, 2012). However, the uptake of Mg by plant root is affected by plant ages, and Mg^2+^ uptake amount per root volume tend to decrease with plant maturation (Kobayashi and Tanoi, 2015). Hence, under conditions of low soil availability and at the time of crop demand nutrient in urgent, foliar application of Mg can be better than soil amendments (Neuhaus et al., 2014). The roles of foliar application of Mg in alleviating Mg deficiency, improving crop yields and quality attributes have been reported for tea, maize, and wheat (Obatolu, 1999; Dordas, 2009; Ceylan et al., 2016; Wolf et al., 2019). There were also reports that foliar application of MgSO_4_·7H_2_O with a level range of 1–5% was commonly practiced to alleviate metabolic disorders and to improve grain yields and quality in many crops (Borowski and Michałek, 2010; Rehman et al., 2018). In this research, foliar Mg application increased rice yields by 7.94% (*P* < 0.05) at four sites under high soil ex-Mg conditions, and the incremental yield was mainly due to the increase in 1,000-grain weight and spikelet number per panicle. However, the intrinsic mechanisms of foliar Mg application to increase rice yield at Mg-sufficient soil still need to be further explored in our next study.

In conclusion, the results of this study showed that the ex-Mg concentrations in the 0–20 cm and 20–40 cm soil layers of Heilongjiang Province paddy fields averaged 282 and 243 mg kg^−1^, respectively. In the 0–20 cm soil layer, the abundant and exceptionally abundant levels accounted for 75%, which was 55.3% in the 20–40 cm layer. Furthermore, the ex-Mg distributions were regionally dependent, with the western part having the highest values in both the 0–20 cm and 20–40 cm layers. Although the ex-Mg contents of the Heilongjiang paddy fields were relatively high, there was no significant effect of soil-applied Mg on rice yields. Foliar application of Mg could significantly increase 1,000-grain weights and spikelet numbers per panicle and thereby increase rice yield.

## Data Availability Statement

The original contributions presented in the study are included in the article/Supplementary Material, further inquiries can be directed to the Corresponding Author.

## Author Contributions

XP, CY, and ZL designed the research. XHL and ZL collected the soil samples. XHL, QH, YC, YD, DL, XYL, and LS did the field experiments. ZL and QH wrote the manuscript. XP and BJ revised the manuscript. MN and XYL edited the language. All authors approved the final manuscript.

## Conflict of Interest

The authors declare that the research was conducted in the absence of any commercial or financial relationships that could be construed as a potential conflict of interest.
