# Supplementary material for: Magnesium Fertilization Affected Rice Yields in Magnesium Sufficient Soil in Heilongjiang Province, Northeast China
Source: Front Plant Sci. 2021 May 11;12:645806. doi: 10.3389/fpls.2021.645806 (PMC8144475; doi:10.3389/fpls.2021.645806)
Supplement: Supplementary file 1 [file Data_Sheet_1.docx]

**Table S1 Effect of soil pH on ex-Mg concentration in 0-20 soil layer. S.D.=Standard Deviation; C.V.=Coefficient of Variation.**

| pH | No. of samples | Ex-Mg（mg·kg^-1^） | S.D. | C.V. |
| --- | --- | --- | --- | --- |
| <5.5 | 54 | 225 | 71 | 0.32 |
| 5.5-6.5 | 75 | 256 | 130 | 0.51 |
| 6.5-7.5 | 30 | 301 | 143 | 0.48 |
| >7.5 | 20 | 510 | 149 | 0.29 |

**Table S2 K/Mg and Ca/Mg of 0-20cm soil layers in different regions of Heilongjiang Province.** **S.D.=Standard Deviation; C.V.=Coefficient of Variation.**

| Property | Region | No. of samples | Average | Minimum | Maximum | S.D. | C.V. |
| --- | --- | --- | --- | --- | --- | --- | --- |
| K/Mg | Songnen Plain | 32 | 0.45 | 0.08 | 2.53 | 0.47 | 1.04 |
|  | South-central | 59 | 0.72 | 0.14 | 1.59 | 0.26 | 0.36 |
|  | Sanjiang Plain | 88 | 0.67 | 0.28 | 2.77 | 0.36 | 0.54 |
| Ca/Mg | Songnen Plain | 32 | 15.22 | 4.03 | 88.18 | 17.08 | 1.12 |
|  | South-central | 59 | 13.73 | 5.21 | 33.51 | 4.43 | 0.32 |
|  | Sanjiang Plain | 88 | 10.03 | 4.82 | 21.81 | 3.54 | 0.35 |
